# Measles Virus-Induced Immune Amnesia and SARS-CoV-2 Evolution

**DOI:** 10.3390/pathogens13030226

**Published:** 2024-03-05

**Authors:** Giovanni Di Guardo

**Affiliations:** General Pathology and Veterinary Pathophysiology, Veterinary Medical Faculty, University of Teramo, Località Piano d’Accio, 64100 Teramo, Italy; gdiguardo@unite.it

An interesting article recently published in *Science Translational* Medicine reports a variable risk of persistent COVID-19 among patients affected by different immunodeficiency conditions. The aforementioned paper’s authors also suggest that suppression of both B and T cell responses is associated with the highest risk of a prolonged SARS-CoV-2 infection [1].

Noteworthy, measles virus (MeV) is a highly lymphotropic pathogen infecting long-lived bone marrow plasmacells. By doing so, MeV may cause a prominent drop in the host’s preexisting antibody response against other infectious agents [2]. This will obviously result in a lower resistance to natural infection as well as in a diminished protection offered by vaccine immunization against the aforementioned pathogens. 

Such a peculiar, MeV-induced mechanism of immune amnesia could also putatively affect SARS-CoV-2 evolution, considering also the viral variants which have already shown a high propensity to escape the immunity conferred by natural infection and/or vaccination [3]. Consequently, the global success achieved against COVID-19 thanks to mass vaccine immunization could turn out to be even more adversely affected.

Regretfully, following a significant drop in children’s vaccination campaigns throughout the COVID-19 pandemic, a skyrocketing increase in measles disease cases has been observed in 2023 in Europe (including Italy) and, to a lesser extent, in extra-European Countries compared with 2022 [4]. This reduction in the European pediatric population’s active immunization had already resulted in an 18% increase in estimated disease cases and in a 43% increase in measles fatalities in 2022 compared with 2021, according to a report released last November by the World Health Organization [5]. This should be a top priority in the global fight against an infectious disease causing over 100,000 deaths worldwide, with mass vaccinations against measles between 2000 and 2021 having saved approximately 57 million lives [5].

In conclusion, an extensive measles vaccine coverage of the general pediatric population is urgently needed on a global scale, with international and national Health Authorities putting their best efforts and encouragement forward in this direction.
